# Navigating the Giant: A Case Series of Giant Choledochal Cysts in Infants Managed Laparoscopically

**DOI:** 10.7759/cureus.108748

**Published:** 2026-05-12

**Authors:** Greeshma Suresh, Surajit Das, Pranav Yadav, Sarita Chowdhary, Umang K Agrawal

**Affiliations:** 1 Pediatric Surgery, Institute of Medical Sciences, Banaras Hindu University, Varanasi, IND; 2 General Surgery, Institute of Medical Sciences, Banaras Hindu University, Varanasi, IND; 3 Department of General Surgery, Employees State Insurance Corporation (ESIC) Medical College and Hospital, Varanasi, IND; 4 Medicine, Nalanda Medical College and Hospital, Patna, IND

**Keywords:** biliary tract anomalies, giant choledochal cyst, hepaticoduodenostomy, laparoscopic cyst excision, pediatric minimally invasive surgery

## Abstract

The incidence of giant choledochal cysts (CDCs) in infants is the rarest of rare, and poses unique surgical challenges due to limited working space and close proximity to vital hepatobiliary structures. With advancements in minimally invasive surgery, laparoscopic excision has become the preferred approach. We present a case series of four infants diagnosed with giant CDCs who underwent laparoscopic cyst excision with biliary reconstruction at our institution. All four patients presented with progressively increasing abdominal distension and were diagnosed using ultrasonography followed by magnetic resonance cholangiopancreaticography. The cyst sizes ranged from 8 cm to 18 cm. Laparoscopic cyst excision with hepaticoduodenostomy was successfully performed in all patients. Technical modifications such as percutaneous cyst decompression and traction using stay sutures were utilized to facilitate safe dissection. The mean operative time was 222.5 (182-273) minutes; mean hospital stay was 8.5 (7-12) days. The follow-up period ranged from three to 16 months. Postoperative recovery was uneventful in three patients. This series demonstrates the feasibility and safety of minimally invasive surgery for giant CDCs in infants when performed with appropriate technical modifications.

## Introduction

Choledochal cysts (CDC) are congenital anomalies of the hepatobiliary system characterized by cystic dilatation of the extrahepatic or intrahepatic biliary tree. Relatively uncommon, but occurs more frequently in Asian populations, with an incidence of approximately one in 13,500 to 20,000, with a strong female predominance. The most widely accepted hypothesis involves an anomalous pancreaticobiliary junction, which results in reflux of pancreatic enzymes into the bile duct, leading to chronic inflammation, weakening of the bile duct wall, and progressive dilatation of the biliary tract [1].

CDCs are most commonly classified according to the Todani classification system, which categorizes the cysts based on their anatomical location and morphology. Type I cysts, involving fusiform or cystic dilatation of the extrahepatic bile duct, represent the most common subtype encountered in clinical practice [2,3]. Giant CDCs are cysts exceeding 10cm in maximum diameter.

The clinical presentation varies with age. Infants frequently present with abdominal distension or a palpable abdominal mass, whereas older children and adults may present with the classical triad of abdominal pain, jaundice, and abdominal mass. Initial evaluation is usually performed with ultrasonography, while magnetic resonance cholangiopancreaticography (MRCP) remains the imaging modality of choice for definitive diagnosis and anatomical delineation of the biliary tree [4].

Complete surgical excision of the cyst with restoration of biliary drainage (hepaticojejunostomy with Roux-en-Y reconstruction) is the treatment of choice, due to the risk of complications such as recurrent cholangitis, pancreatitis, biliary obstruction, and malignant transformation [4]. In recent years, minimally invasive surgery (MIS) has been gaining acceptance for the management of CDC because of its advantages, including improved visualization, reduced postoperative pain, shorter hospital stays, and superior cosmetic outcomes [5].

Despite these advantages, laparoscopic management of giant CDCs, particularly in infants, remains technically challenging due to limited working space for port placement and also the close proximity of the cyst to critical vascular and pancreatic structures. In this study, we present our experience with laparoscopic management of four infants with giant CDCs and the technical modifications that facilitated safer surgical excision.

## Case presentation

Case 1

**Figure 1:**
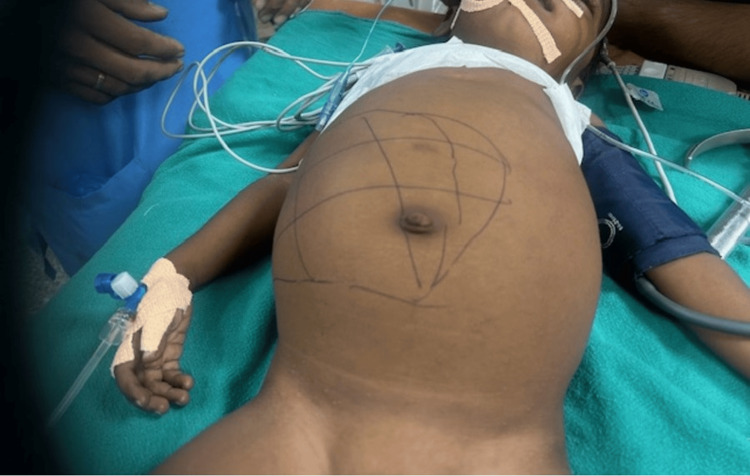
Clinical photograph demonstrating abdominal distension in an infant caused by a giant choledochal cyst presenting as an upper abdominal mass. Image from Case 1.

An eight-month-old female presented with progressively increasing abdominal distension for two months, not associated with feed intolerance, vomiting, jaundice, fever, or altered bowel habits. On examination, the vitals were stable, upper abdomen was distended with a large palpable cystic mass occupying the epigastric and right upper quadrants, with smooth margins, non-tender nature and some mobility, only in the transverse plane (Figure 1). Baseline laboratory investigations and MRCP findings are summarised in Table 1. The child underwent laparoscopic CDC excision with hepaticoduoodenostomy with an uneventful postoperative course, was started orally on postoperative day (POD) 3, and was discharged on POD 6, with a hospital stay of seven days.

**Table 1:** Summary of cases Abbreviations: TLC: Total Leukocyte Counts, PT: Prothrombin Time, INR: International Normalised Ratio, SGPT: Serum Glutamic Pyruvic Transaminase, SGOT: Serum Glutamic Oxaloacetic Transaminase, U/L: Units/Litre

Case	1	2	3	4	Reference Range
Age (months)	4	9	11	13	-
Sex	Female	Female	Male	Female	-
Hemoglobin (g/dl)	11.5	9.6	10.8	12.1	12.5-16.5
TLC (cells/mm³)	7.23*10³	16.98*10³	6.78*10³	6.46*10³	4000-11000
Platelets (10³/mm³)	143	154	101	121	150-450
Total bilirubin (mg/dl)	0.35	12.9	1.11	1.02	0.2-1.2
Direct bilirubin (mg/dl)	0.21	10.6	0.89	1	<0.3
PT (seconds)/INR	11/1.11	16/1.4	11/1.03	10/1.13	12-16/<1.4
SGPT (U/L)	32	87	38	24	<45
SGOT (U/L)	36	53	40	31	<35
Type of CDC (Todani)	1	1	1	1	-
Size (max diameter)	8*10	10.3*11.4	15.8*12.4	11.4*12.2	-
Duration of surgery (minutes)	182	273	225	210	

Case 2

A nine-month-old female presented with progressive abdominal distension associated with intermittent abdominal pain, fever, and jaundice for one week, associated with decreased appetite. On examination, the infant appeared icteric but was hemodynamically stable, with a large cystic mass palpable in the right upper quadrant extending towards the epigastrium. Laboratory investigations (Table 1) and ultrasonography of the abdomen demonstrated a large CDC (10 × 11) cm. She was initially managed conservatively for acute cholangitis with intravenous antibiotics (ceftriaxone, metronidazole, and amikacin), with supportive care including intravenous fluids and analgesics. Subsequently, the patient was discharged and scheduled for definitive surgery two weeks later.

MRCP confirmed a Type I CDC (10.3 × 11.4) cm. The patient underwent laparoscopic cyst excision with hepaticoduodenostomy. Intraoperatively, dense adhesions were encountered between the cyst wall, pancreas, and duodenum. During dissection, bleeding from the cyst wall was noted, which was controlled laparoscopically, but it prolonged the mean operative time by about 17.5 minutes. The postoperative course was notable for transient ileus. Enteral feeds could be started on POD 7 and she was discharged on postoperative day 11, with a hospital stay of 12 days in this sitting.

Case 3

An 11-month-old male presented with progressive abdominal distension associated with vomiting and early satiety for one month. On examination, the infant was vitally stable, with a large palpable non-tender mass occupying the epigastric and right upper quadrant measuring approximately 18 × 12 cm, with a smooth surface and cystic consistency. Laboratory investigations are shown in Table 1. The child underwent laparoscopic cyst excision with hepaticoduodenostomy with an uneventful post-operative course. Oral feeding was initiated on POD 3 and he was discharged on POD 6, with a hospital stay of seven days.

Case 4

A 13-month-old female presented with a gradually progressive epigastric mass associated with episodes of abdominal pain for four months. On examination, the infant was hemodynamically stable, with a large cystic mass in the epigastric and right upper quadrants. The mass was smooth, non-tender, and mobile.

**Figure 2:**
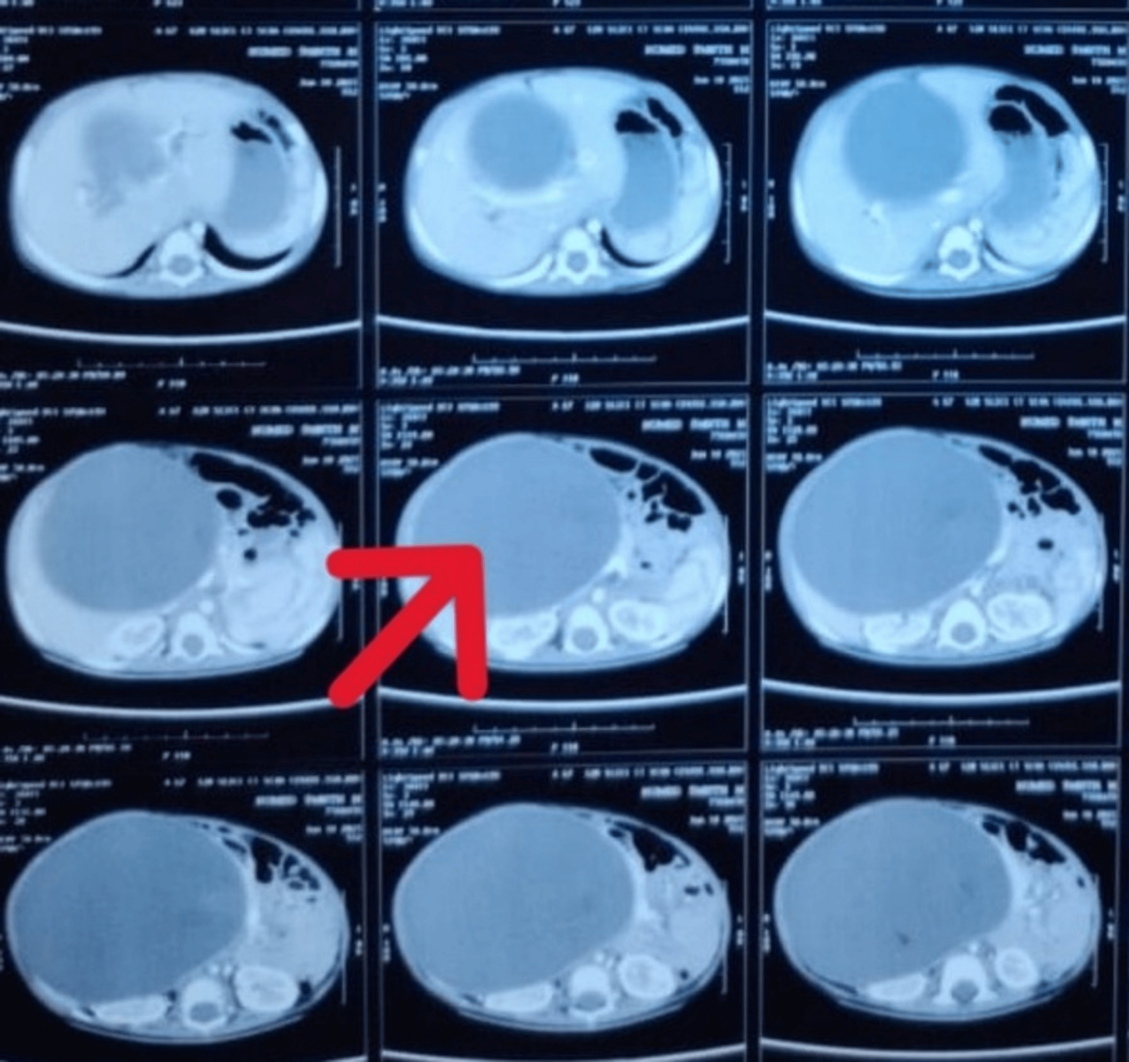
Magnetic resonance cholangiopancreaticography (MRCP) demonstrating a giant choledochal cyst (CDC) (red arrow) Cross sectional view showing a giant cystic lesion occupying nearly the whole of right upper abdomen. Here the red arrow shows the giant cystic lesion, while the different slices of MRCP shows the mass effect leading to displacement of adjacent organs. Image from Case 4.

Laboratory investigations and MRCP are shown in Table 1 and Figure 2. The patient underwent laparoscopic cyst excision with hepaticoduodenostomy. The postoperative course was uneventful, with feeds initiated on POD 4 and discharged on POD 7, with a hospital stay of eight days.

Operative technique

All procedures were performed under general anesthesia with the patient placed in the supine position. After standard painting and draping, pneumoperitoneum was established through the first port inserted at Palmer’s point, which further guided the placement of the remaining ports. A camera port was then placed at the umbilicus, while two additional working ports were positioned in the lumbar regions in a triangulated and ergonomic configuration.

**Figure 3:**
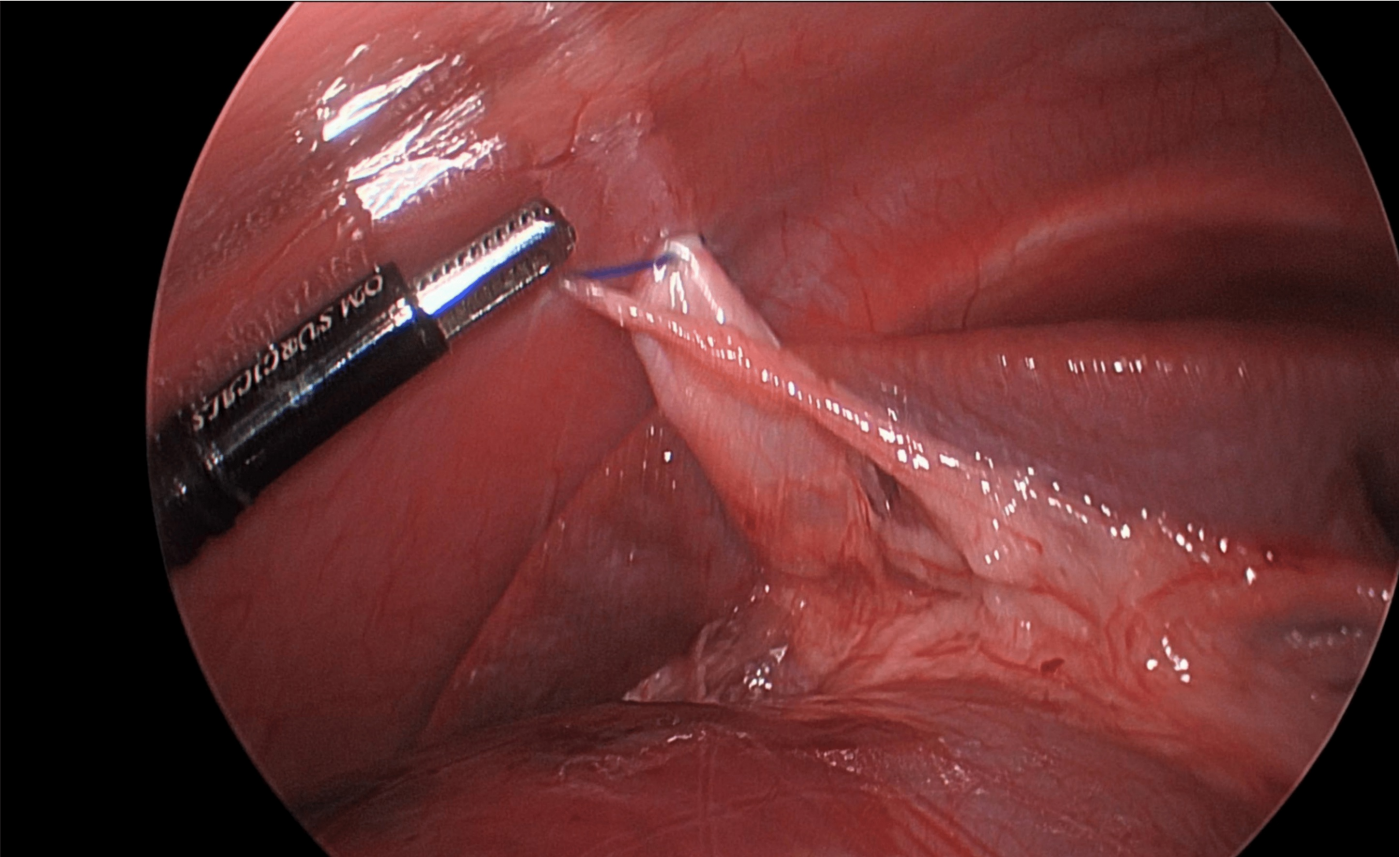
Stay suture placed to aid dissection Intra-operative image from Case 3.

**Figure 4:**
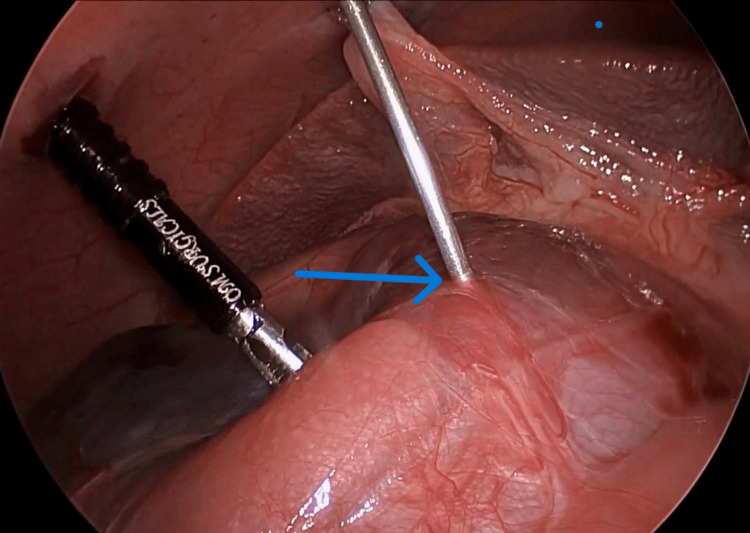
Demonstrates per-cutaneous aspiration of cyst contents for ease of dissection (blue arrow). Image sourced from Case 3.

A percutaneously placed stay suture was used to provide traction on the CDC (Figure 3), which facilitated exposure and allowed controlled manipulation of the cyst during dissection. In cases of giant CDCs, occupying most of the upper abdominal cavity, controlled percutaneous aspiration of cyst contents was performed to decompress the cyst (Figure 4). This significantly increased the working space and improved visualization of surrounding structures.

**Figure 5:**
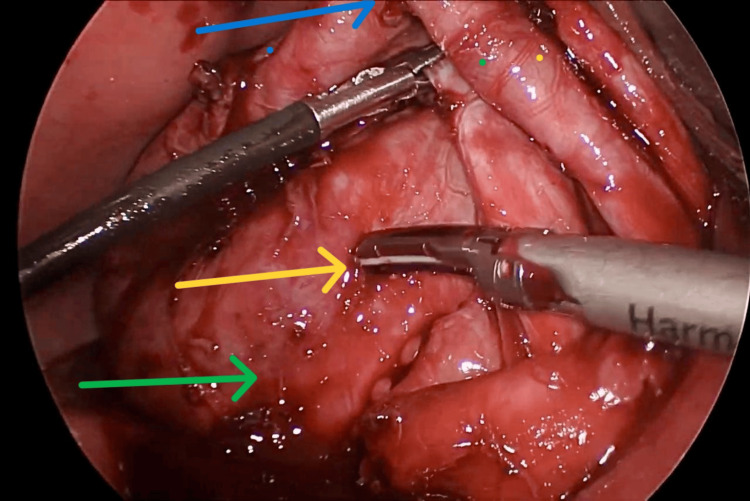
Intra-operative demonstration of the dissection of giant choledochal cyst (CDC) Blue arrow: traction by suture, Yellow arrow: dissection aided by harmonic device, Green arrow: giant CDC. Image sourced from Case 3.

**Figure 6:**
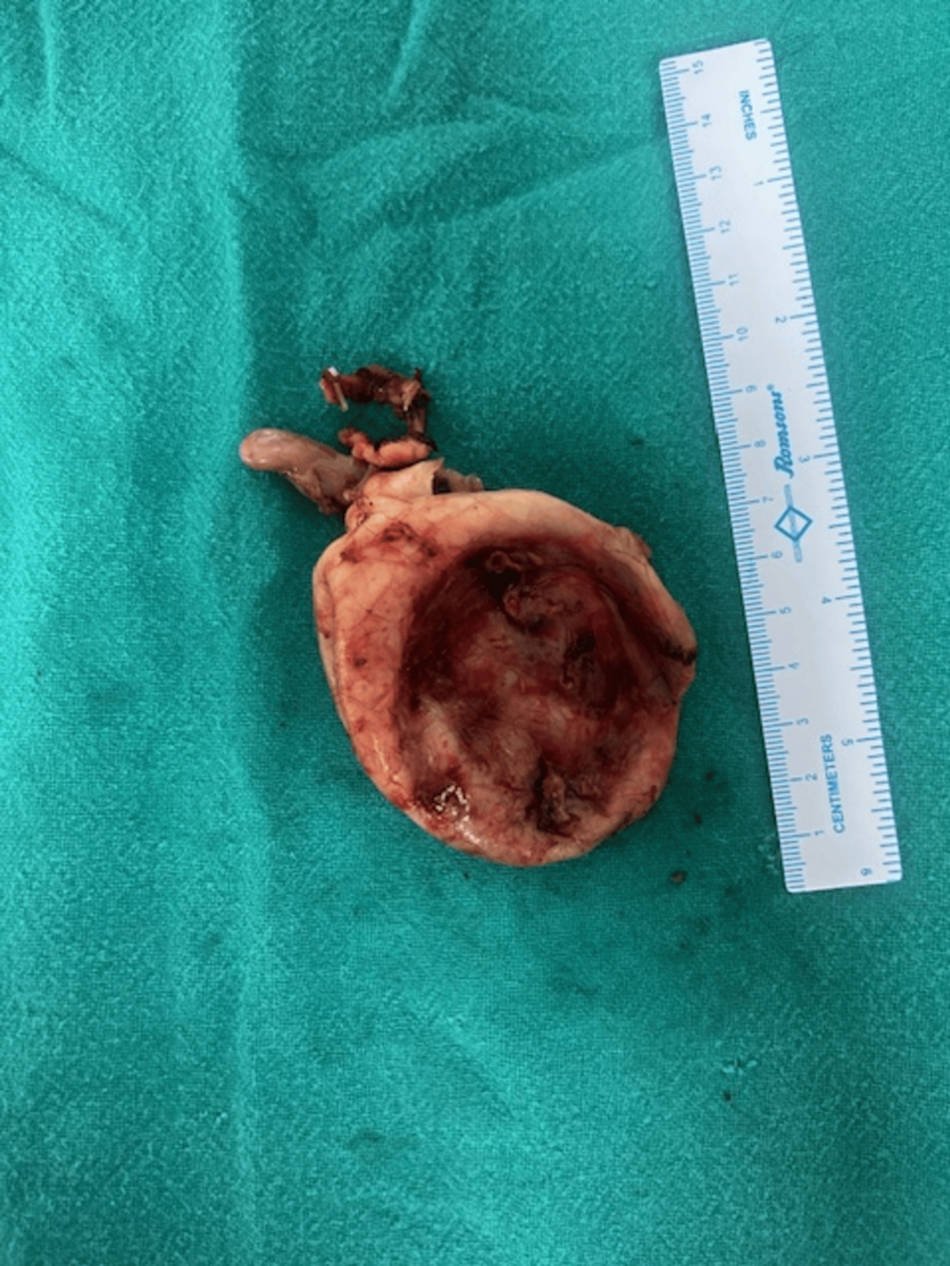
Excised specimen of the giant choledochal cyst following laparoscopic cyst excision Image from Case 4.

Dissection was carried out along the surface of the cyst in a caudocranial and lateromedial direction, carefully separating it from adjacent structures including the duodenum, pancreas, portal vein, and hepatic artery. Cranial dissection was continued up to the common hepatic duct just distal to the confluence of the right and left hepatic ducts. The gallbladder and cystic duct were excised along with the cyst as part of the surgical specimen (Figure 5). The mobilized cyst was temporarily placed over the superior surface of the liver to facilitate reconstruction. The specimen was then retrieved via the umbilical port (Figure 6).

The second part of the duodenum was mobilized up to the porta hepatis to allow a tension-free biliary anastomosis. Patency of the right and left hepatic ducts was confirmed, and any biliary sludge present was flushed with normal saline. Biliary reconstruction was performed using hepaticoduodenostomy with interrupted 5-0 polyglactin sutures. A right subhepatic drain was placed near the anastomosis to monitor for any postoperative bile leak. The specimen was retrieved through the umbilical port site, and the port sites were closed.

## Discussion

CDCs are congenital anomalies of the biliary tract characterized by cystic dilatation of a part or the entire biliary tree. Despite the rarity, it occurs more frequently in Asian populations, with an estimated incidence of approximately one in 13,500 to one in 20,000 compared with about one in 100,000 in Western populations. A female predominance has been consistently reported in most epidemiological studies [1]. Giant CDCs are defined as cysts exceeding 10 cm in maximum diameter. Though the exact etiology of CDCs remains incompletely understood, one of the most widely accepted theories involves an anomalous pancreaticobiliary junction, which leads to reflux of pancreatic enzymes into the bile duct, further leading to chronic inflammation and progressive weakening and dilatation of the bile duct wall [1,2].

The clinical presentation varies depending on the age at diagnosis. Infants frequently present with abdominal distension or a palpable abdominal mass, while older children and adults may present with the triad of abdominal pain, jaundice, and abdominal mass. Ultrasonography is usually the initial screening modality. However, MRCP remains the investigation of choice as it provides an excellent delineation of biliary anatomy and helps in determining the type of CDC as per the Todani classification. Untreated CDCs may lead to complications including recurrent cholangitis, pancreatitis, biliary obstruction, stone formation, and malignant transformation. Therefore, complete surgical excision of the cyst with restoration of biliary drainage remains the standard treatment [4].

Traditionally, CDC excision was performed using an open surgical approach. However, with the advancement of MIS, laparoscopic cyst excision has become increasingly popular. The laparoscopic approach offers advantages such as superior visualization, magnification of the operative field, reduced postoperative pain, shorter hospital stays, faster recovery, and improved cosmetic outcomes [6]. Despite these advantages, laparoscopic management of giant CDCs, particularly in infants, can be technically challenging, as it may occupy most of the upper abdominal cavity, resulting in limited working space. In addition, giant cysts may be densely adherent to adjacent structures such as the duodenum, pancreas, portal vein, and hepatic artery, increasing the complexity of dissection [7,8]. In such cases, open technique was traditionally preferred.

In our experience, certain technical modifications proved particularly helpful in facilitating safe laparoscopic excision of giant CDCs. Firstly, the initial placement of the port at Palmer's point, then the use of a stay suture for traction, allowing better control of the cyst during dissection and improved exposure of the operative field. This technique helped in maintaining adequate tension on the cyst wall and facilitated safer separation from surrounding structures. Then, the controlled decompression of the cyst through percutaneous aspiration significantly reduced the cyst size and increased the available working space. This step was especially useful in infants where the limited intra-abdominal space poses a challenge during MIS. While hepaticojejunostomy with Roux-en-Y reconstruction remains the traditional approach, hepaticoduodenostomy has been described as a viable alternative in select cases [9]. 

Similar technical challenges and operative considerations in MIS in CDC surgery have been highlighted in previous studies [10,11]. Overall, our experience demonstrates that with appropriate surgical expertise and careful intraoperative technique, laparoscopic excision of giant CDC in infants is feasible and safe.

## Conclusions

Giant CDC in infancy is uncommon and may present significant technical challenges during MIS due to the limited working space and proximity to vital hepatobiliary structures. Our experience demonstrates that laparoscopic cyst excision with hepaticoduodenostomy is feasible and safe in infants with giant CDC when performed by a team of experienced surgeons, with some technical modifications such as initial port placement at Palmer’s point, application of stay sutures, and controlled cyst decompression, facilitating safe dissection.
